# Structural genome variation drives adaptation of the xylose-fermenting yeast *Scheffersomyces stipitis* to lignocellulosic hydrolysates

**DOI:** 10.1007/s10577-026-09813-6

**Published:** 2026-09-02

**Authors:** Samuel Vega-Estévez, Andrew Armitage, Bruce S. Dien, Patricia J. Slininger, Alessia Buscaino

**Affiliations:** 1 Kent Fungal Group, School of Biosciences, Division of Natural Sciences, University of Kent Canterbury, Kent, CT2 7NZ UK; 2 Natural Resources Institute, University of Greenwich, Kent, ME4 4TB UK; 3 Bioenergy Research Unit, National Center for Agricultural Utilization Research, USDA, Agricultural Research Service, 1815 N. University, Peoria, IL 61604 USA; 4 Present Address: Quadram Institute Bioscience, Norwich Research Park, Norwich, NR4 7UQ UK; 5 Present Address: Centre for Microbial Interactions, Norwich Research Park, Norwich, NR4 7UG UK; 6 Present Address: University of East Anglia, Norwich, NR4 7TJ UK

**Keywords:** Structural genome variation, Minichromosome formation, Chromosomal rearrangements, Adaptive evolution, Bioethanol production, Industrial yeast adaptation

## Abstract

Second-generation (2G) bioethanol from lignocellulosic feedstocks is a sustainable alternative to fossil fuels. However, its production is constrained by the poor performance of industrial microbes in hydrolysates that are generated during biomass pretreatment. *Scheffersomyces stipitis* is a native xylose fermenting yeast and a promising platform for 2G bioethanol production, and adaptive evolution under hydrolysate stress has yielded strains with enhanced performance. However, the chromosomal basis of this adaptation is unknown. Here, we demonstrate that chromosome scale structural variation, rather than point mutations, underlies the improved phenotype of the evolved strains. By integrating long- and short-read genome sequencing, we identify two major chromosomal rearrangements in the top performing isolate: a reciprocal translocation between chromosomes 1 and 2 that disrupts the NUDIX hydrolase gene *YSA1*, and the formation of a mitotically stable 175 kb minichromosome derived from chromosome 5. Functional analyses show that disruption of *YSA1* enhances xylose utilisation and ethanol yield, while the minichromosome contributes to improved performance in hydrolysate conditions. These findings provide direct evidence that balanced rearrangements and minichromosome formation can be selected during prolonged stress and can generate adaptive phenotypes. Taken together, our study establishes genome reorganisation as a key driver of adaptation in *S. stipitis*.

**Supplementary Information:**

The online version contains supplementary material available at https://doi.org/10.1007/s10577-026-09813-6.

## Introduction

Genomes are continually shaped by evolutionary pressures imposed by environmental and industrial stressors. Populations with greater genetic variation adapt more rapidly than those with limited diversity (Bomblies and Peichel 2022). This variation arises through both small-scale mutations and extensive structural chromosomal changes (Sui et al. 2020).

Small-scale mutations in protein-coding genes include single-nucleotide variants (SNVs), that can modify gene regulation or protein function, influencing cellular pathways. In contrast, large chromosomal alterations reshape genome architecture and can give rise to simple or complex phenotypes (Dunham et al. 2002; Tang et al. 2021). These structural changes include balanced rearrangements, such as translocations and inversions, and unbalanced variants, such as whole-chromosome or segmental aneuploidies (Weischenfeldt et al. 2013). Balanced variants can disrupt transcriptional networks or alter DNA topology, whereas unbalanced changes lead to gains or losses of genetic material, resulting in copy number variation (CNV) and imbalanced protein levels (Harewood and Fraser 2014; Harewood et al. 2017; Wang et al. 2020). Although the fitness consequences of extensive chromosomal changes remain poorly understood, they are widespread in microbial populations and play a central role in adaptation under natural selection (Sheltzer and Amon 2011). For example, large chromosomal rearrangements, including CNVs, complex aneuploidies and balanced rearrangements, have been identified in laboratory, industrial and wild yeast isolates (Peter et al. 2018; O'Donnell et al. 2023), and they drive drug adaptation in pathogenic fungi such as *Candida albicans* and *Cryptococcus neoformans* (Selmecki et al. 2006; Stone et al. 2019).

2G bioethanol derived from lignocellulose has an enormous potential to reduce our societal carbon footprint, both as a renewable transport fuel and as a platform chemical. Unlike ethanol produced from starch or sugar based feedstocks, lignocellulosic ethanol overcomes concerns associated with competition with food production. (Mohr and Raman 2015). Herbaceous biomass consists primarily of glucans (cellulose), xylans and lignin (Robak and Balcerek 2018). During pretreatment, these polymers release mixed sugar streams, glucose from cellulose and xylose from xylan, so economic production of ethanol requires microorganisms that ferment both sugars. A major technological barrier to commercialisation of lignocellulose conversion is that distillers’ *S. cerevisiae* yeast, the workhorse of global ethanol production, cannot ferment xylose. In contrast, *Scheffersomyces stipitis*, a non-pathogenic haploid yeast, displays the highest known native capacity among yeast to convert both glucose and xylose selectively to ethanol with balanced redox cofactors and considerable resilience to inhibitors, making it a strong candidate for 2G bioethanol production (Bruinenberg et al. 1984; Slininger et al. 1985, 2006, 2009; du Preez et al. 1989; Sims et al. 2010).

*S. stipitis* belongs to the CTG clade of fungi, in which the CTG codon is translated as serine rather than leucine (Papon et al. 2014). Its haploid genome comprises eight chromosomes encoding approximately 6,000 protein-coding genes and contains complex regional centromeres enriched in the retrotransposons Tps5a, Tps5b and Tps5c, together with large retrotransposon derivative (LARD) elements (Jeffries et al. 2007; Coughlan and Wolfe 2019; Vega-Estévez et al. 2021). Although *S. stipitis* isolates share highly conserved coding sequences, they exhibit substantial variation in genome organisation, with retrotransposon-rich regions acting as hotspots for chromosome rearrangements and genome plasticity (Vega-Estévez et al. 2021).

Despite its biotechnological promise, *S. stipitis* still requires substantial improvement as a biocatalyst for industrial 2G bioethanol production (Agbogbo and Coward-Kelly 2008; Wu et al. 2023). This is because it preferentially consumes glucose over xylose and is highly sensitive to ethanol during xylose fermentation, largely because xylose reductase and xylitol dehydrogenase are repressed under ethanol stress (Slininger et al. 2011). This limitation is particularly important because xylose is a major sugar in herbaceous lignocellulosic hydrolysates. Its utilisation can be inhibited by the accumulation of ethanol produced during prior glucose metabolism, which may cause a diauxic lag when yeast shifts from glucose to xylose consumption. Without a preinduction phase, poor utilisation of xylose in mixture with glucose results in prolonged diauxic lag and reduced ethanol yields (Slininger et al. 2011).

Fermentation of hydrolysates also impose additional stresses. Diluted acid or hot water pretreatment generates inhibitory compounds such as furan aldehydes and acetic acid and typically yields media with very low nitrogen availability (Jeffries 2008). Ammonia fiber expansion (AFEX) avoids furan formation but introduces high ammonia levels and reduces sugar availability (Chundawat et al. 2020). Even with improvements in enzyme engineering and ammonia recycling (Chundawat et al. 2020), AFEX hydrolysates still contain acetic acid and phenolic compounds that impair fermentation (Casey et al. 2010; Chen et al. 2020). Because of this complexity, adaptive laboratory evolution (ALE) under defined selective pressures has proven highly effective for generating *S. stipitis* isolates with improved fermentation performance under industrial conditions without prior knowledge of the underlying genetic basis of the trait. In a previous study, selection was designed to favour natural variants capable of fermenting xylose with minimal diauxic lag in the presence of ethanol and inhibitor-rich xylose hydrolysates, resulting in isolates with markedly enhanced industrial performance (Slininger et al. 2015). However, previous attempts to identify the genetic basis of enhanced *S. stipitis* performance using short-read Illumina sequencing failed to reveal mutations that could explain the evolved phenotype, leaving its molecular basis unresolved (Smith and Quinlan 2008).

To address this gap, we combined Illumina short-read sequencing with Oxford Nanopore long-read sequencing to comprehensively characterise both small mutations and chromosomal-scale variation in two superior bioethanol-producing isolates, *sup-2* (NRRL-Y50859; USA patent 9,297,027) and *sup-4* (NRRL-Y50861; USA patent 9,297,027) obtained after long-term adaptation in hydrolysates prepared from switchgrass and corn stover, as well as in defined medium containing high ethanol concentrations (Slininger et al. 2015). Our results indicate that small-scale mutations make only a limited contribution to the improved performance, whereas both balanced and unbalanced chromosomal rearrangements are the main determinants of the industrially relevant phenotype.

## Material and methods

### Yeast strains and growth conditions

Table S1 contains a list of strains, and Table S2, a list of primers used in this study. Strains were obtained from the Agricultural Research Service (ARS) Collection, run by the National Center for Agricultural Utilization Research (NCAUR, formerly Northern Regional Research Laboratory (NRRL) Peoria, IL, and their identities were confirmed by sequencing of the 26S rDNA [D1/D2 domain] (Villa-Carvajal et al. 2006) using primers AB798 and AB799. Flask cultures of *S. stipitis* were incubated at 30 ⁰C mixed at 200 rpm and filled 20% w/v with yeast extract/peptone/d-glucose (YPD) medium supplemented with uridine (0.08 g/L) and adenine hemisulfate (0.05 g/L). Solid media were prepared by adding 2% w/v agar.

### Fermentation analysis and ranking performance

#### Preparation of switchgrass hydrolysate (SGH)

Switchgrass hydrolysates were prepared from cultivar Kanlow N1 harvested post-frost from Mead, NE, USA, which had been milled to pass through a 2 mm screen. Switchgrass was pretreated at 20% w/w solids by mixing 20 g dry weight of biomass and 2.4 g ADM (Archer Daniels Midland) Toasted Nutrisoy Flour (Product 063160) with 8 mL of 0.936% (v/v) sulfuric acid solution and 0.3 g Pluronic F-268 surfactant. After pretreatment, the product was adjusted to pH 4.5 by adding 7.14 mL of 15% Ca(OH)_2_ solution and 4.5 mL of 1 M citric acid buffer (pH 4.8) directly into each vessel and then tumbling 15 min in the Labomat. Pretreatment hydrolysates were transferred to 250 mL Pyrex screw cap bottles for saccharification. To each bottle, 2.7 mL of CTec2 and 0.5 mL of HTec2 enzymes (Novozyme) were added then caps tightened, and hydrolysates were incubated approximately 72 h at 50 °C and 175 rpm to saccharify. Resulting hydrolysates were amended with 2.36 g/L urea, adjusted to pH 6.2, then filter sterilized using 0.2 μm Nalgene filter units and stored at 4 °C. Composition of SGH with soy flour and urea amendments was given previously (Slininger et al. 2015, 2023).

#### Strain performance on SGH

For comparing kinetic phenotypes of isolates, precultures were inoculated by sterile loop from 24–48 h YM agar purity plates to 30 mL Optimal Defined Medium (ODM) with 50 g/L xylose in 50 mL flasks (Bellco silicone sponge plug closures). Culture media were previously described (Slininger et al. 2015). The ODM precultures were incubated 150 rpm (1” orbit) 48 h at 25 °C and then used to inoculate 30 mL challenge cultures on 50%(v/v) SGH to OD_620_ ~ 0.5, which were similarly incubated. The 72 h challenge cultures were pelleted and concentrated to OD_620_ 50, then used to inoculate 24 mL test cultures on 100% SGH to OD_620_ 0.5 (initial pH 6.2). Cultures were incubated in 50 mL flasks as above and sampled daily to track cell growth (OD_620_) and concentrations of sugar and ethanol using high performance liquid chromatography (HPLC), as previously described (Slininger et al. 2015). Each strain was tested in duplicate in each of two experiments. In one experiment isolates were cultivated in SGH at full strength (100%), and in a second experiment they were cultivated in 80% v/v SGH (prepared by diluting SGH with water). Two primary performance factors, ethanol yield and xylose uptake rate, were used to evaluate improvements in xylose fermentation. These were the same criteria used previously to rank and select the superior evolved isolates generated by adaptive laboratory evolution (Slininger et al. 2015). The secondary performance factors were ethanol production rate and glucose uptake rate. Although adaptive laboratory evolution primarily selected for improved xylose fermentation, these secondary factors were included to ensure that improvements in xylose utilisation were not accompanied by reduced glucose fermentation or ethanol productivity. Isolates were ranked in both 80% and 100% SGH media using a dimensionless Relative Performance Index (RPI). RPI was calculated from the mean (X_avg_) and standard deviation (s) of each data set, as follows: RPI = (2 + F) × 100/4, where F = (X- X_avg_)/s. The factor *F* is the standard score (z-score), which represents the number of standard deviations by which an observation differs from the dataset mean. For normally distributed data, most observations fall between − 2 and + 2, corresponding to RPI values between 0 and 100. The multiplier “100/4″ scales the transformed values to this range, with 0 representing the lowest and 100 the highest relative performance (Slininger et al. 2015). Overall RPI values were calculated by averaging the RPIs of the two primary performance factors. Secondary performance factors were analysed separately to confirm that improvements in xylose fermentation were not accompanied by reduced glucose utilisation or ethanol production.

### Genetic analysis of superior strains

#### Contour-clamped homogeneous electric field (CHEF) electrophoresis and Southern blotting

Intact yeast chromosomal DNA was prepared as previously described (Vega-Estévez et al. 2021). Briefly, cells were grown overnight, harvested from an OD_600_ ≈ 7 culture by centrifugation, and embedded in agarose plugs by resuspending the pellet in 300 µl of 1% low-melt agarose (Bio-Rad, catalog no. 1613112) prepared in 100 mM EDTA with 25 µl of 10 mg/ml Zymolyase 100 T (Amsbio, catalog no. 120493–1). The plugs were suspended in Zymolyase reaction buffer (100 mM EDTA, 100 mM Tris, 1% β-mercaptoethanol) solution and incubated overnight at 37 ⁰C. After that, the plugs were washed in 50 mM EDTA twice and resuspended in proteinase K reaction buffer (100 mM EDTA, 0.2% SDS, 1%, 0.2 mg/ml proteinase K) and incubated for two nights at 50 ⁰C. Plugs were then washed with 50 mM EDTA and stored at 4 ⁰C until run. Chromosomes were separated in a 1% Megabase agarose gel (Bio-Rad) in 0.5 × TBE using a CHEF DRII apparatus. Running conditions for karyotype visualization were 60- to 120 s switch at 6 V/cm for 12 h, followed by a 120- to 300 s switch at 4.5 V/cm for 12 h at 14 ⁰C. For mChr5 chromosome visualization the running conditions were switched to 60 to120 s, 6 V/cm for 12 h and 120 to 300 s, 4.5 V/cm for another 12 h. Chromosomes were visualized by staining the 0.5 × TBE gel with ethidium bromide (0.5 μg/ml) for 30 min, followed by destaining in water for 30 min. Images were captured using a Syngene GBox Chemi XX6 gel imaging system.

For Southern Blot the DNA from CHEF gels were transferred overnight to a Zeta-Probe GT Membrane (Bio-Rad, catalog no. 162–0196) in 20 × SSC and cross-linked using UV (150 mJ). Probing and detection of the DNA were conducted as previously described (Vega-Estévez et al. 2021). Briefly, probes were generated by PCR incorporation of DIG-11-dUTP into target sequences according to the manufacturer’s instructions (Roche). Chromosome 5 and mChr5 were targeted using the primers AB994 and AB995. The membrane was incubated with the probe overnight at 42⁰C with DIG Easy Hyb (Roche, catalog no. 11603558001). The DNA was detected with anti-digoxigenin-alkaline phosphatase antibody (Roche, catalog no. 11093274910) and CDP Star Ready-to-Use (Roche, catalog no. 12041677001) according to the manufacturers’ instructions.

### Genome sequencing

The genomes of *sup-2* (NRRL-Y50859) and *sup-4* (NRRL-Y-50861) isolates were sequenced by Illumina short-read and Oxford Nanopore long-read sequencing technologies. DNA was extracted from an overnight culture using a Qiagen genomic tip 100/G kit (Qiagen, catalog no. 10243) according to manufacturer’s protocol. For long-read sequencing, MinION sequencing (Oxford Nanopore, Oxford, UK) was performed on a DNA library prepared from size-selected gDNA. DNA fragments greater than 30 kb were selected using a Blue Pippin (Sage Science) and concentrated using AmPure beads. From this, a DNA library was prepared using a 1D ligation sequencing kit (SQK-LSK108) and run on the Oxford Nanopore MinION flow-cell FLOMIN 106D. The same gDNA extract was also used for the preparation of Illumina libraries. In this case, the DNA was sheared using a Covaris M220 with microTUBE-50 (catalog no. 520166) and size selected using the Blue Pippin (Sage Science). The library was constructed using a PCR-free kit with NEBNext End Repair (E6050S), NEB Next dA-tailing (E6053S), and Blunt T/A ligase (M0367S) New England Biolabs modules. Sequencing was performed on a MiSeq benchtop analyzer (Illumina) using a 2 × 300-bp PE (MS-102–3003) flow cell.

### Bioinformatic analyses

Genome assembly and bioinformatic analyses are documented in the GitHub repository: https://github.com/harrisonlab/pichia/tree/master and were executed on HPC systems across NIAB East Malling Research, University of Kent and University of Greenwich. Job submission scripts via SunGridEngine are called within GitHub documentation but are documented within organisational private repositories. As such, the details of core run commands executed for each program are detailed in the Supplementary Information.

### Gene deletion and yeast transformation

*YSA1* deletion in the NRRL Y-7124 parental strain was achieved via homologous recombination using a linear YSA1-NATR DNA fragment containing a nourseothricin resistance gene flanked by 500 bp regions homologous to YSA1. The YSA1-NATR fragment was generated by overlap extension PCR. First, the 500 bp upstream and downstream of YSA1 were PCR amplified from NRRL Y-7124 genomic DNA using primers AB1209 + AB1213 and AB1210 + AB1215, generating products 1 and 2. The nourseothricin resistance gene was amplified from pHA_NAT plasmid (AB17) using primers AB1214 + AB1216 containing overhangs annealing to products 1 and 2. Products 1–3 were stitched by PCR without primers, followed by a second PCR with primers AB1209 and AB1210.Yeast were transformed by freeze-transformation (Passoth et al. 2003) and plated on YPD with 200 µg/ml nourseothricin. Correct integrants were identified by PCR using primers AB659 and AB1211.

### Identification and validation of the chromosome 5-derived minichromosome

Illumina short-read and Oxford Nanopore long-read sequencing data were mapped to the *S. stipitis* reference genome as described above. Copy number variation was identified from sequencing depth by comparing read coverage between the parental and evolved strains. Oxford Nanopore reads mapping to the chromosome 5 CNV were manually inspected using IGV to identify reads containing *S. stipitis* telomeric repeat sequences (5′-ATGGATCTTTTCACGTCTTGCGGT-3′) and to assess whether reads linked the amplified region to adjacent chromosome 5 sequences.

To validate the predicted chromosome termini, we designed a PCR assay using a chromosome 5-specific primer immediately proximal to the CNV(AB1015) and a primer complementary to telomeres (AB1018).

The presence of an independent chromosome was confirmed by CHEF electrophoresis followed by Southern blotting using a chromosome 5-specific probe.

### Chromosome loss assay

Seven colonies containing the minichromosome mChr5 were grown in 5 ml YPD for 15 h. Cells were plated on YPD agar at low density. After 48 h at 30 °C, 10 colonies per plate were assessed for mChr5 presence by PCR using primers AB1015 and AB1018. Actin PCR with primers AB800 and AB801 served as control. The experiment was performed in triplicate, analysing 210 colonies total. mChr5 absence was confirmed on a selection of colonies by Southern blotting, amplifying the probe with primers AB994 and AB995.

## Results

### Adaptation to industrial conditions leads to genomic reorganisation and centromeric transposon changes in *S. stipitis*

**Fig. 1:**
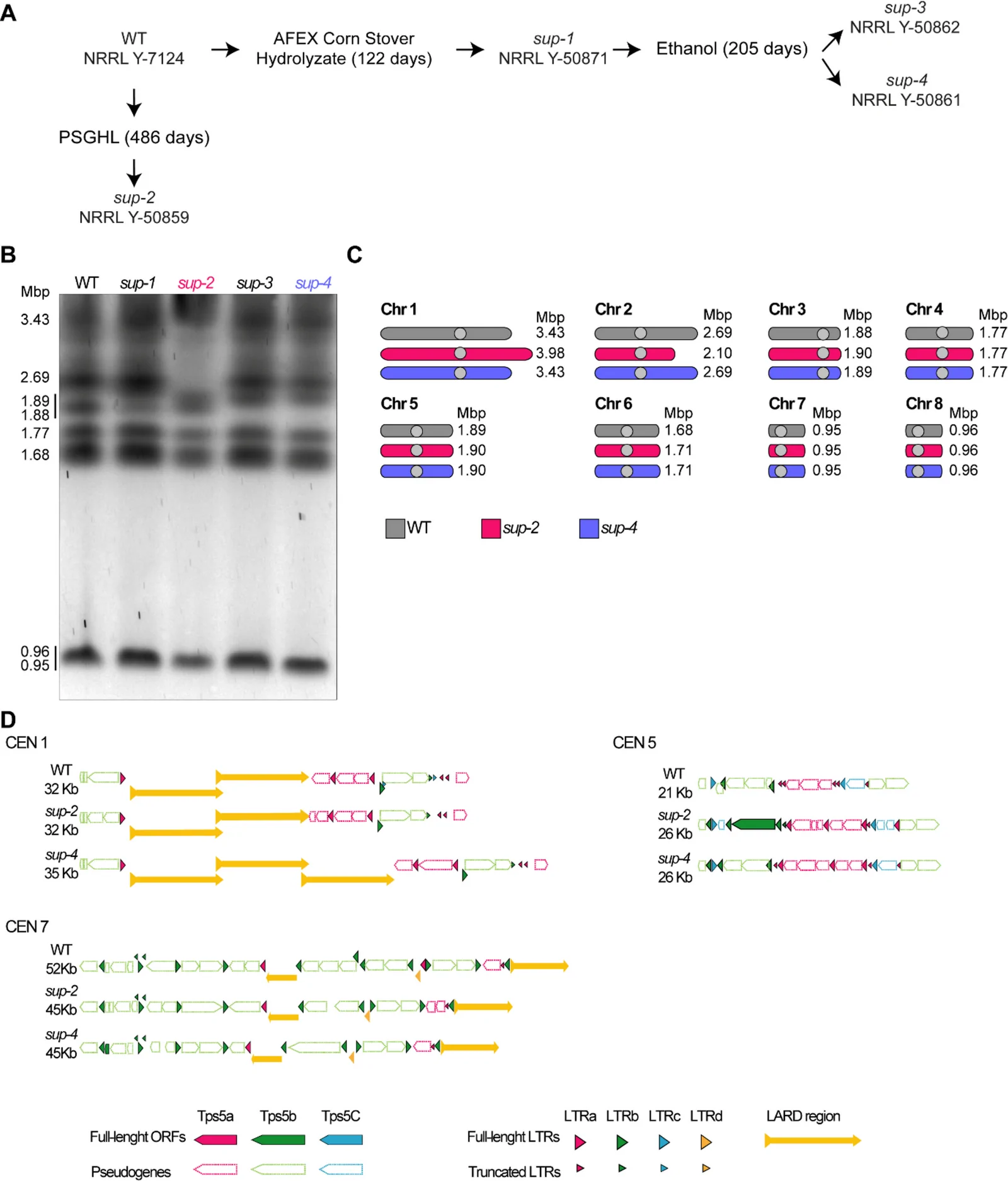
Karyotypic and centromeric organization of evolved *S. stipitis* strains. (**A**) Schematic representation of the ALE experiments leading to the selection of strains *sup-1*, *sup-2*, *sup-3*, and *sup-4*. The selection media and the duration of each selection (in days) are indicated. (**B**) Karyotype analysis of *S. stipitis* isolates adapted to industrial hydrolysates. Chromosomes were separated by contour-clamped homogeneous electric field (CHEF) electrophoresis. The wild-type strain (WT; NRRL Y-7124) is shown alongside four evolved isolates, with *sup-2* and *sup-4 *highlighted in magenta and blue as the focus of this study. The sizes of the eight chromosomes are indicated. (**C**) Schematic representations of chromosomes from WT (NRRL Y-7124), *sup-2*, and *sup-4* strains. Chromosome (Chr) number and size (Mbp) are shown. (**D**) Organization of centromeres CEN1, CEN5, and CEN7 in WT (NRRL Y-7124), *sup-2*, and *sup-4* strains. The number and positions of full-length transposons, long terminal repeats (LTRs), and large retrotransposon derivative (LARD) elements are indicated

To identify genetic changes associated with adaptation to hostile environments within *S. stipitis*, we focused on four superior (*sup*) bioethanol producer strains derived from the wildtype (WT) strain NRRL Y-7124 during a previous Adaptive Laboratory Evolution (ALE) study (Slininger et al. 2015): *sup-1*, exposed to ammonia fiber expansion (AFEX) hydrolysates of corn stover for 122 days; *sup-2*, exposed to xylose-rich, glucose-poor dilute-acid pretreated switchgrass hydrolysate liquor (PSGHL) for 486 days; *sup-3* and *sup-4*, exposed to AFEX (122 days) then sequential passaging in increasing ethanol for 205 days (Fig. 1A).

We first tested whether the *sup* strains had acquired a distinct genomic organisation by contour-clamped homogeneous electric field (CHEF) electrophoresis, a technique that separates chromosomes according to size. Strain *sup-2* differed in chromosomal organisation compared to the WT strain, whereas *sup-1*, *sup-3*, and *sup-4* displayed less pronounced differences (Fig. 1B).

The resolution of CHEF-gel electrophoresis is insufficient to identify the genomic changes that may have occurred during the evolution experiments. To overcome this limitation, the genomes of the superior strains *sup-2* and *sup-4* were sequenced by combining short-reads Illumina sequencing with long-read Oxford Nanopore Technologies (ONT). The high accuracy of Illumina sequencing enabled reliable identification of point mutations and small indels, whereas the long read length of ONT sequencing enabled the resolution of large-scale structural rearrangements (Goodwin et al. 2016). The strain *sup-2* was chosen due to its different karyotype and because it was the only strain adapted to the most challenging environment: PSGHL media that is rich in xylose and poor in glucose (Slininger et al. 2015). The strain *sup-4* was selected because it was one of two strains sequentially adapted to two stresses: AFEX hydrolysate and high ethanol concentration (Slininger et al. 2015). The assembled genome demonstrated that *sup-2* and *sup-4*, like the parental WT strain, have a core genome organised into eight chromosomes (Fig. 1C and Fig. S2). Previously, we showed that different *S. stipitis* natural isolates have distinct genomic organisations and that retrotransposons are prominent drivers of this diversity (Vega-Estévez et al. 2021). While non-centromeric transposons were unchanged in abundance and localisation, we detected differences in centromeric retrotransposon organisation, specifically *sup-2* and *sup-4* CEN-5 and CEN-7 showed Tps5a copy number changes and *sup-4* CEN-1 had extra transposon-derived LARD elements (Fig. 1D). Therefore, transposable elements were drivers of genome diversity as the yeast adapted to these hostile environments.

### Small genetic mutations are unlikely drivers of enhanced ethanol production

Although variation in centromere size can influence chromosome segregation, it is unlikely to explain the superior performance of the *sup-2* and *sup-4* strains, because centromeric regions lack protein-coding genes (Coughlan and Wolfe 2019) and commonly tolerate extensive accumulation of transposable elements without measurable fitness effects (Klein and O’Neill 2018). To test whether coding-sequence changes contributed to the evolved phenotypes, we aligned Illumina reads from both strains to the parental reference genome and identified strain-specific variants. This analysis detected no nonsense mutations and no small insertions or deletions (< 20 bp), indicating that experimental evolution may not have frequently generated or did not select for highly disruptive mutations in protein-coding regions. Instead, we identified seven missense mutations, three shared by both evolved strains and four detected only in *sup-4* (Table 1). The presence of shared variants suggests that these mutations may have been present in the ancestral population prior to selection, although independent origin cannot be excluded. Structural models indicated that none of the missense changes are predicted to compromise protein folding (Fig. S1). Specifically, in *sup-4*, PICST_49608 (382 aa), an uncharacterised protein containing two AlphaFold-predicted domains, carries a substitution in domain 1 that is not predicted to affect its structure. PICST_62836, an MCP-domain signal transduction protein (632 aa), harbours a T481N substitution with no predicted structural consequences. PICST_33084 (907 aa) is predicted to be largely intrinsically disordered, except for a 126-aa α-helix-rich region, and the mutation lies outside this structured domain. Finally, PICST_73970 (1045 aa), a putative RNA cytidine acetyltransferase, carries a K79N substitution that is likewise not predicted to affect protein folding. Collectively, these findings suggest that the protein-coding mutations identified are unlikely to account for the enhanced phenotypes of *sup-2* and *sup-4*.

**Table 1:** List of SNPs associated with *sup-2* and *sup-4* superior strains

ID	Gene Name	Chr	WT (aa)	*sup-2* (aa)	*sup-4* (aa)	Type	Description
PICST_46361	OCA1	Chr5	H120	Y120	Y12	Missense	Tyrosine/serine phosphatase
PICST_31552		Chr4	D29	E29	E29	Missense	PAPA-1 containing proteins
PICST_49608		Chr7	R23	I23	I23	Missense	Hypothetical Protein
PICST_59269		Chr5	E54	E54	E54	Missense	Hypothetical Protein
PICST_62836		Chr6	T481	N481	N481	Missense	Hypothetical Protein
PICST_33084		Chr6	N409	I409	I409	Missense	Hypothetical Protein
PICST_83970		Chr8	K79	N79	N79	Missense	RNA cytidine acetyltransferase

### A chromosomal translocation disrupts the NUDIX hydrolase Ysa1 in the superior *sup-2* strain

**Fig. 2:**
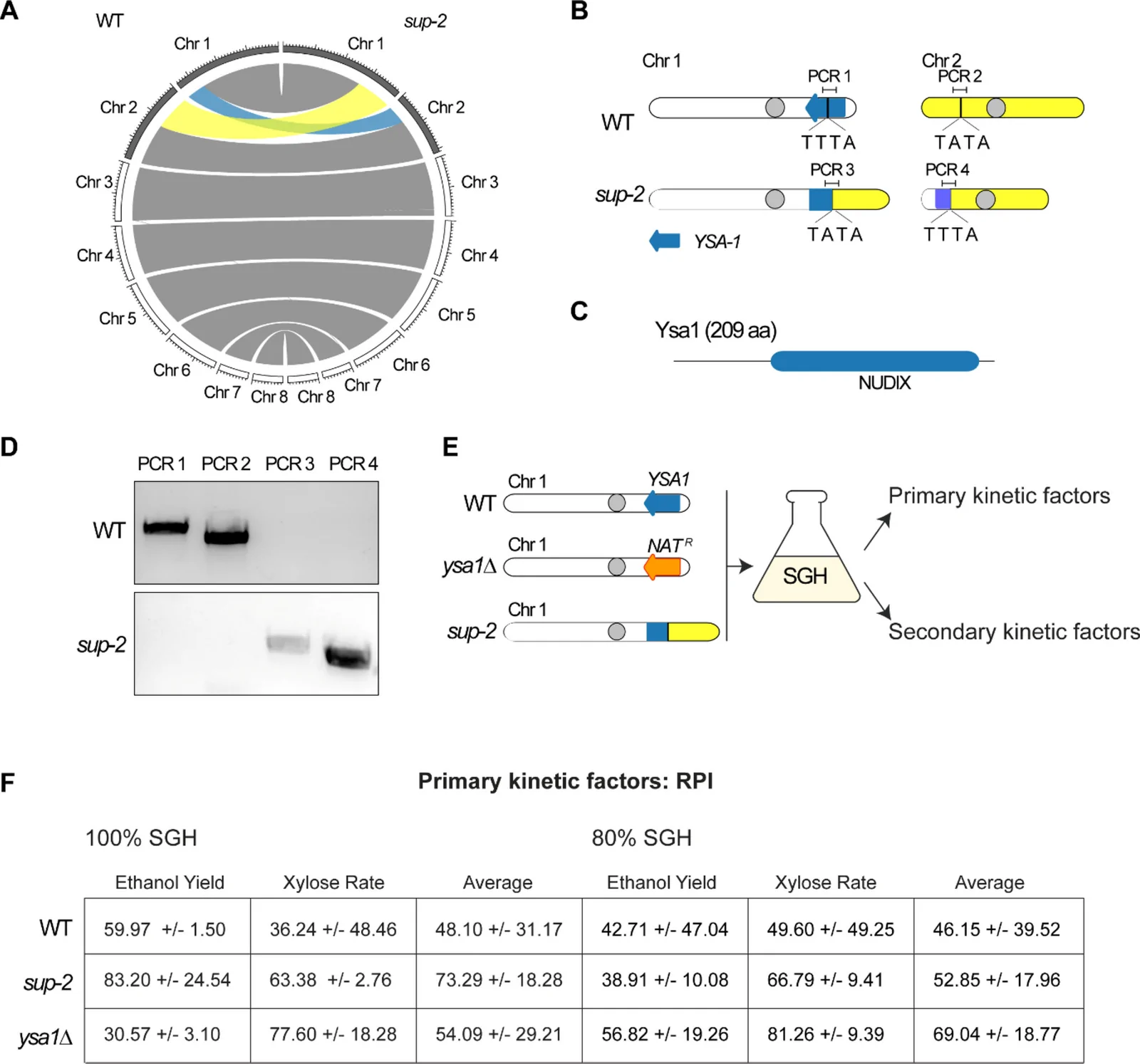
A chromosomal translocation disrupts the NUDIX hydrolase gene *YSA1* in the superior *sup-2* strain. (**A**) Circos plot illustrating macrosynteny between the wild-type (WT; left) and *sup-2* (right) strains, with chromosome (Chr) numbers indicated. The reciprocal translocation between chromosomes 1 and 2 is highlighted in yellow and blue. (**B**) Schematic representations of chromosomes 1 and 2 in WT and *sup-2* strains. The sequence at the translocation breakpoint is shown, together with the position of the *YSA1* gene and the PCR amplicons (PCR1, PCR2, PCR3, and PCR4) used for diagnostic assays. (**C**) Agarose gel electrophoresis of amplification products from PCR1, PCR2, PCR3, and PCR4 in WT and *sup-2* strains (**D**) Schematic representation of the Ysa1 protein showing total length and the position of the NUDIX hydrolase domain. (**E**) Experimental design used to assess fermentation performance of WT, *ysa1Δ*, and *sup-2* strains. (**F**) Relative Performance Index (RPI) for the primary performance traits (ethanol yield and xylose uptake rate) in WT, *sup-2*, and *ysa1Δ* strains under either 100% or 80% switchgrass hydrolysate (SGH) conditions (*n* = 4)

Because only a limited number of small-scale mutations were detected during experimental evolution, and none could plausibly explain the superior phenotypes of *sup-2* or *sup-4*, we hypothesised that selection favoured large-scale genomic alterations. ONT sequencing did not detect any structural variation in *sup-4* (Fig. S2); however, it revealed a translocation between chromosome 1 and chromosome 2 (Chr1-Chr2) in *sup-2* that truncates the previously uncharacterized gene *PICST_53805* (Fig. 2A and B). We validated this rearrangement by PCR (Fig. 2C). *PICST_53805* encodes a protein containing a NUDIX hydrolase domain that we have designated *S. stipitis* Ysa1, based on its orthology to *S. cerevisiae Ysa1* (Fig. 2D and Fig. S3)*.* It has been shown that deletion of *S. cerevisiae YSA1* leads to increased NADPH availability and improved xylose utilization (Dunn et al. 1999; Tong et al. 2009), suggesting a potential functional link between disruption of *PICST_53805* and the enhanced phenotype observed in *sup-2.* To assess whether loss of *YSA1* function contributes to the *sup-2* phenotype, we generated an *ysa1Δ* mutant in the parental background and compared its fermentation performance with that of the WT and *sup-2* strains. We cultured the WT, *sup-2*, and *ysa1Δ* strains in SGH and quantified the primary perfomance factors (ethanol yield and xylose uptake rate) which were previously used to select the superior strains during experimental evolution (Slininger et al. 2015) (Fig. 2E). We also measured secondary performance factors (ethanol production rate and glucose uptake rate) which were not used as isolate selection criteria but were monitored to ensure that improvements in xylose fermentation did not compromise overall fermentation performance. (Slininger et al. 2015) (Fig. 2E). We calculated the Relative Performance Index (RPI) to rank strains based on these parameters. When challenged with 100% SGH, the average primary RPI indicated improved performance in both *sup-2* and *ysa1Δ* relative to the WT parent at a 85% confidence level (*sup-2*: 73.29 ± 18.28%; *ysa1Δ*: 54.09% ± 29.21%; WT Y7124: 48.10% ± 31.17%) (Fig. 2F and Table S3B). We repeated the fermentation experiments in 80% (v/v) SGH to enable two-way analysis of variance with isolate and SGH strength as variables. This analysis confirmed the results obtained in 100% SGH: at 80% SGH the WT strain had a lower RPI for primary performance factors (46.15 ± 39.52%) than *sup-2* (52.85 ± 17.96%) and *ysa1Δ* (69.04 ± 18.77%) at a 83% confidence leve (Fig. 2F and Table S4B). The lack of a significant isolate × SGH-strength interaction (Table S5) permitted pooling of the 100% and 80% SGH data, thereby increasing the degrees of freedom from four to eight per isolate and improving the power of the ANOVA. This procedure also streamlined the analysis and simplified interpretation.These results confirm that the *sup-2* and *ysa1Δ* strains consistently outperform the WT parent for primary-factor performance under both SGH conditions, while secondary-factor performance is comparable across all engineered strains (Table S5).

Thus the genetic changes in *sup-2* and *ysa1Δ* produced greater improvements in the primary xylose fermentation traits targeted during adaptive laboratory evolution (Slininger et al. 2015). Together, these results indicate that the chromosome rearrangement selected during experimental evolution contributed, at least in part, to the superior performance of *sup-2* through disruption of *YSA1*.

### Formation of a mitotically stable minichromosome during selection of superior *S. stipitis* bioethanol producer isolates

**Fig. 3:**
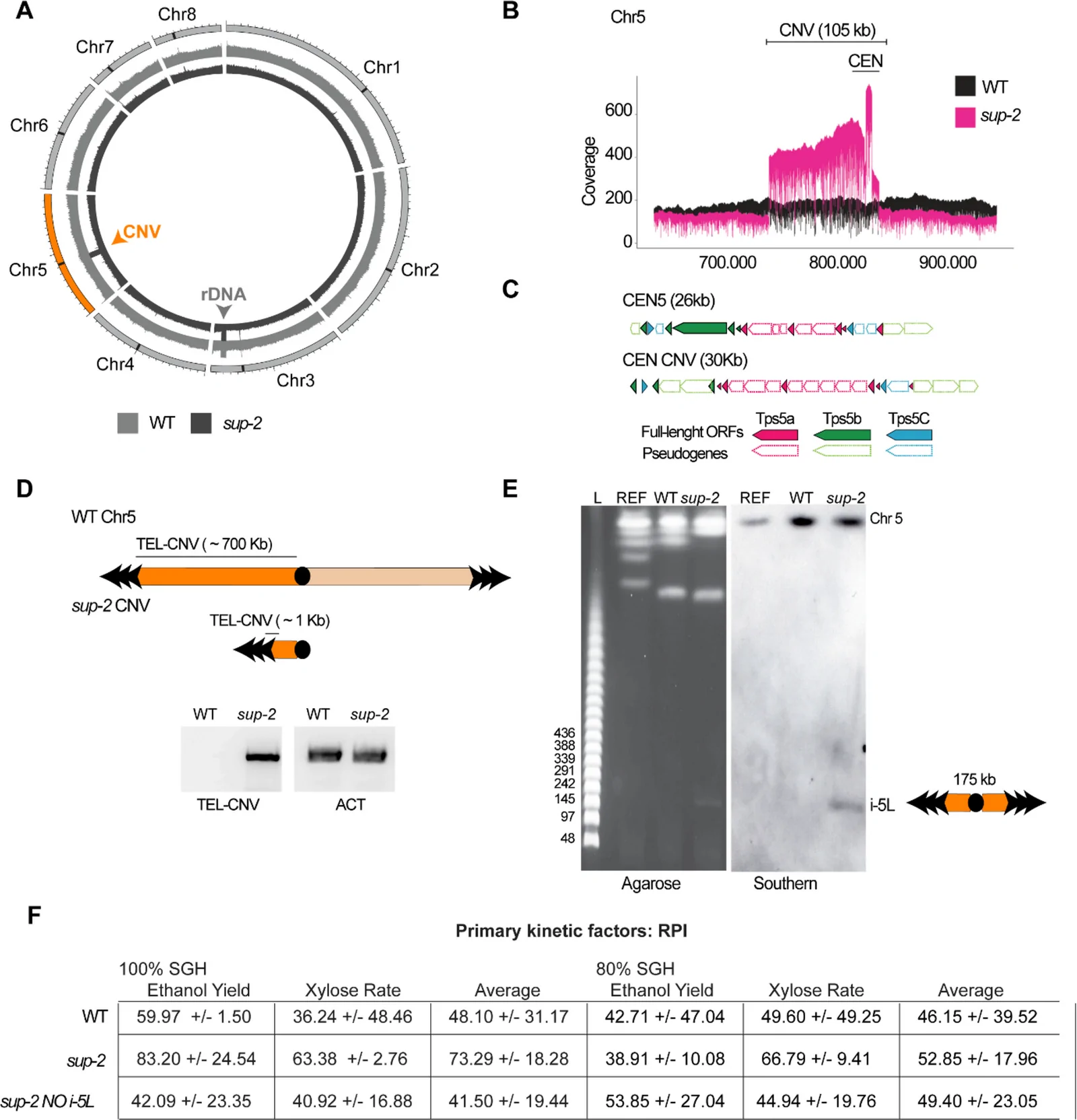
Selection of a mitotically stable minichromosome during adaptation of *S. stipitis* to industrial hydrolysates. (**A**) Circular plots showing normalized read coverage of the WT (light grey) and *sup-2* (dark grey) strains across all chromosomes (Chr). A region of increased copy number on the left arm of chromosome 5 (Chr5) in *sup-2* is highlighted in orange and labeled as a copy number variation (CNV). (**B**) Long-read sequencing coverage across Chr5. Read depth (y-axis) is shown for WT (black) and *sup-2* (magenta) strains, with genomic coordinates indicated on the x-axis. The position of the centromere (CEN) is indicated. (**C**) Schematic representation of centromere (CEN) organisation in the endogenous chromosome 5 and in the CNV identified in *sup-2*. (**D**) Schematic representations of full-length Chr5 and the minichromosome derived from the left arm of Chr5 (i-5L), showing the positions of the centromere (CEN), telomeric repeats (Tel), and predicted genes. Diagnostic PCR (TEL-CNV) and control (ACT) assays in WT and *sup-2* strains are shown below. (**E**) Left: karyotype analysis of *S. stipitis* strains by CHEF electrophoresis. L, lambda DNA ladder; REF, reference strain NRRL Y-11545; WT, parental strain NRRL Y-7124; *sup-2*, superior strain carrying the minichromosome (i-5L +). Right: Southern blot of CHEF-separated chromosomes hybridized with a Chr5-specific probe, confirming the presence of the minichromosome in *sup-2*. The final structure of i-5L is indicated. (**F**) Relative Performance Index (RPI) for the primary performance traits (ethanol yield and xylose uptake rate) of WT and *sup-2* strains with and without i-5L under 100% or 80% switchgrass hydrolysate (SGH) conditions. Values represent means ± SD from four biological replicates; the average RPI is shown for each condition

Read-depth analysis of Illumina and ONT sequencing identified a second large scale chromosomal alteration in the *sup-2* genome. We detected an approximately 100 kb CNV on Chr5 comprising a ~ 30 kb region spanning the centromere (CEN5) and an adjacent ~ 70 kb region containing 24 protein-coding genes (Fig. 3A and B). Sequencing coverage across most of the CNV was approximately two-fold higher than in the parental strain. In contrast, the CEN5 region has higher apparent coverage (Fig. 3B). Inspection of individual ONT reads suggested that the centromeric region associated with the CNV is larger than the endogenous CEN5 (~ 30 kb versus ~ 26 kb) and differs in its repeat organisation, which likely accounts for the increased coverage (Fig. 3C). A second region of elevated sequencing coverage was present in both the parental and evolved strains (Fig. 3A). This corresponds to the ribosomal DNA (rDNA) locus, a highly repetitive genomic region whose copy number is naturally variable.

We next examined ONT reads spanning the amplified region. We identified reads containing the *S. stipitis* telomeric repeat sequence (5′-ATGGATCTTTTCACGTCTTGCGGT-3′) at the predicted end of the duplicated fragment. To validate this observation, we designed a PCR assay using one primer located immediately proximal to the centromeric CNV and a second primer complementary to telomeric regions. Although the chromosome 5-specific primer also anneals to the endogenous chromosome, amplification from the native locus would require extension across approximately 700 kb and is therefore not expected under the PCR conditions used (Fig. 3D). Thus, amplification is only possible if the centromeric CNV is directly capped by telomeric repeats, as predicted by the ONT reads. This primer pair amplified the expected product exclusively in the evolved strain (Fig. 3D). The presence of both telomeric repeats and CEN5 suggested that the amplified DNA forms an independent linear chromosome rather than a duplicated region within Chr5. To test this hypothesis, we separated intact chromosomes by CHEF electrophoresis and hybridised them with a chromosome 5-specific probe. Southern blot analysis detected an additional linear chromosome of approximately 175 kb in *sup-2* that was absent from the parental strain (Fig. 3E). This chromosome was substantially larger than the ~ 100 kb CNV identified by sequencing. Because the amplified region spans CEN5, the most parsimonious model that reconciles the sequencing and molecular data is duplication of the left arm of Chr5 around the centromere to generate an isochromosome, which we named i-5L (Fig. 3E).

We next quantified the mitotic stability of i-5L. We propagated seven independent *sup-2* cultures overnight in rich medium, isolated single colonies and screened them using the i-5L PCR assay (Fig. S2A). Nine of the 210 colonies analysed lacked the diagnostic PCR product, corresponding to a chromosome loss rate of approximately 4.3 ± 1.4% (Fig. S2B). CHEF electrophoresis and Southern blotting independently confirmed the loss of i-5L in these isolates (Fig. S2C), demonstrating that the chromosome is transmitted through mitosis but can be lost in the absence of selection.

Gene annotation identified 24 genes within the duplicated chromosome arm that are predicted to be present in three copies in *sup-2* (one copy on chromosome 5 and two copies on i-5L; Table S6). These genes encode proteins involved in metabolism, cellular signalling, information storage and processing, together with several proteins of unknown function. To examine whether the presence of i-5L is associated with differences in fermentation performance, we compared the original *sup-2* isolate (containing i-5L) with three isolates that had lost the minichromosome (*sup-2* NO i-5L). We cultured those strains in SGH and calculated RPI based on primary performance factors (ethanol yield and xylose uptake rate) and secondary performance factors (ethanol production rate and glucose uptake rate). In 100% SGH, *sup-2* isolates carrying i-5L showed higher average primary RPI values (73.29 ± 18.28%) than isolates lacking i-5L (41.50 ± 19.44%), indicating that loss of the minichromosome results in reduced performance at an 85% confidence level (Fig. 3F and Table S3B). In 80% SGH, the *sup-2* isolate (retaining i-5L) again showed higher average primary RPI (52.85 ± 17.96%) than isolates lacking i-5L (49.40 ± 23.05%), albeit the magnitude of improvement was smaller, which would be consistent with the reduced inhibitor challenge (Fig. 3F and Table S4B). When data from both SGH concentrations were considered together, isolates that had lost i-5L consistently exhibited lower RPI values for the primary performance factors, whereas secondary performance factors were less affected (Table S5). Thus, long-term exposure to hostile environments led to the formation and selection of a minichromosome that improved bioethanol production from industrial hydrolysates.

## Discussion

Understanding how organisms respond and adapt to selective pressures is a central question in evolutionary biology. At the same time, defining the genomic basis of improved microbial performance in lignocellulosic hydrolysates is critical for advancing 2G bioethanol production. These dual motivations make adaptive laboratory evolution of industrial yeasts a powerful framework for studying how genome architecture contributes to complex traits. Although *S. stipitis* is among the most efficient native xylose-fermenting yeasts, previous studies of evolved strains were unable to identify clear genetic determinants underlying improved performance in industrial hydrolysates (Smith et al. 2008). By combining long- and short-read genome sequencing with functional genetic and phenotypic analyses, we investigated the genomic basis of adaptation in two independently evolved strains, *sup-2* and *sup-4*, obtained after more than 12 months of adaptive laboratory evolution in industrial hydrolysates (Slininger et al. 2015). Our results show that these strains followed distinct evolutionary trajectories.

### Genome structural variation as a major driver of adaptation in sup-2

In *sup-2*, we identified two major structural genomic alterations that together explain much of its improved fermentation phenotype. The first is a balanced reciprocal translocation between chromosomes 1 and 2 that disrupts the NUDIX hydrolase gene *YSA1*. In *S. cerevisiae*, Ysa1 functions as an ADP-ribose pyrophosphatase, and its loss redirects carbon flux toward the pentose phosphate pathway and increases NADPH availability, thereby enhancing redox balance and resistance to oxidative and chemical stress (Dunn et al. 1999; McLennan 2006; Lee et al. 2014). Increased NADPH availability is also expected to benefit xylose metabolism, which relies on tightly coupled redox reactions. Consistent with this metabolic role, we found that deletion of *YSA1* in the parental *S. stipitis* background improves xylose uptake and fermentation performance, supporting the conclusion that disruption of YSA1 contributes to the adaptive phenotype of *sup-2* under hydrolysate conditions.

The second major genomic alteration in *sup-2* is the formation of the isochromosome i-5L derived from the chromosome 5. This finding adds to growing evidence that large chromosomal rearrangements, including complex aneuploidies, are important mechanisms of adaptation in laboratory, industrial and wild yeast populations, and frequently arise during adaptive laboratory evolution of industrial yeasts (Mangado et al. 2018; O'Donnell et al. 2023; Muenzner et al. 2024; Podgorsek et al. 2025). Because i-5L retains a functional centromere and is capped by telomeric repeats, it behaves as an independent chromosome and it is functionally similar to accessory chromosomes of filamentous fungi (Ma et al. 2010; Goodwin et al. 2011; Croll et al. 2013; D'Ambrosio et al. 2017; Ferree et al. 2024) and adaptive whole-chromosome aneuploidies in budding yeast (Yona et al. 2012), which are transmitted through mitosis and confers a selective advantage. In *sup-2*, i-5L increases the copy number of 24 genes involved in metabolism, signalling and information processing. We hypothesise that the simultaneous increase in gene dosage across multiple pathways enhances metabolic robustness and inhibitor tolerance during growth in lignocellulosic hydrolysates. The functional contribution of i-5L is supported by phenotypic comparisons showing that *sup-2* isolates that lost i-5L during non-selective growth exhibited reduced fermentation performance.

The spontaneous loss of i-5L during growth in the absence of selection (~ 4.3% per generation) indicates that i-5L is mitotically stable but it is not essential for viability and can be lost during cell division. While retained, the duplicated genes on i-5L may provide an opportunity for adaptive mutations to arise independently of chromosome duplication, ultimately reducing the selective advantage of maintaining the chromosome. Together, these observations support the emerging view that aneuploidy provides a transient adaptive solution by increasing gene dosage until more stable compensatory mutations arise, after which the aneuploid state is progressively lost (Kohanovski et al. 2024).

Together, disruption of *YSA1* and formation of the chromosome 5-derived isochromosome i-5L illustrate how large-scale chromosomal rearrangements can rapidly generate adaptive phenotypes by simultaneously altering the dosage of multiple genes.

### Limited coding variation in *sup-4* and potential regulatory adaptation

In contrast to *sup-2*, the enhanced fermentation performance of *sup-4* cannot be readily attributed to large-scale chromosomal rearrangements or to clearly disruptive mutations in protein-coding genes. We identified a distinct centromere organisation in *sup-4*; however, this is unlikely to contribute directly to the improved phenotype because centromeric regions lack protein-coding genes and can tolerate variation in repetitive elements without measurable fitness consequences (Klein and O’Neill 2018; Coughlan and Wolfe 2019). In addition, genome sequencing revealed no nonsense mutations and no small insertions or deletions (< 20 bp) in *sup-4*, arguing against selection of highly deleterious mutations during experimental evolution. Instead, we detected only missense substitutions, three of which were shared between *sup-2* and *sup-4* and four that were specific to *sup-4*. The presence of shared variants suggests that these mutations may have been present in the ancestral population prior to selection, although independent origin cannot be excluded. Structural models indicated that none of the missense changes are predicted to compromise protein function.

The contrasting genomic signatures of *sup-2* and *sup-4* are plausibly explained by their distinct selective histories. The strain *sup-2* had evolved under sustained exposure to dilute-acid hydrolysate, characterized by increasingly high concentrations of fermentation inhibitors and xylose-rich, glucose-poor conditions, whereas *sup-4* experienced an initial phase in AFEX-treated hydrolysate followed by ethanol stress. These environments impose different physiological constraints that are likely favour different adaptive strategies. The comparatively milder and more heterogeneous pressures experienced by *sup-4* may have promoted incremental tuning of gene expression rather than selection for extensive chromosomal rearrangements or coding mutations. One plausible explanation is that adaptation in *sup-4* involves chromatin organisation and epigenetic processes. Changes in chromatin accessibility, histone modifications, nucleosome positioning, or stable transcriptional states can produce heritable shifts in gene expression without altering underlying DNA sequence (Ge and Brickner 2024). In agreement with this hypothesis, stress-induced transcriptional memory and chromatin remodelling have been shown to persist across generations and to facilitate rapid adaptation to fluctuating environments in yeast (D'Urso and Brickner 2014; Audergon et al. 2015; Torres-Garcia et al. 2020). Although we did not directly assess chromatin state or global transcriptional profiles in this study, the paucity of structural and coding variation in *sup-4* is consistent with an adaptive trajectory dominated by regulatory change. Future work integrating transcriptomics and chromatin profiling will be required to determine whether stable regulatory states contribute to the enhanced fermentation performance of this strain.

In summary, this study reveals that long-term adaptation of *S. stipitis* to industrial hydrolysates incurred profound genome restructuring and underscores the central role of genome plasticity in shaping complex industrial phenotypes.

## Supplementary Information

Below is the link to the electronic supplementary material.

## Data Availability

Whole Genome Shotgun projects have been deposited at DDBJ/ENA/GenBank under BioProject PRJNA609885 with the accessions JADGGB000000000 and JADGGC000000000. The versions described in this paper are versions JADGGB010000000 and JADGGC010000000.
