# Supplementary material for: Structural genome variation drives adaptation of the xylose-fermenting yeast Scheffersomyces stipitis to lignocellulosic hydrolysates
Source: Chromosome Res. 2026 Sep 2;34(1):21. doi: 10.1007/s10577-026-09813-6 (PMC13538198; doi:10.1007/s10577-026-09813-6)
Supplement: Supplementary file 1 [file 10577_2026_9813_MOESM1_ESM.docx]

**Supplementary Information (SI)**

Structural genome variation drives adaptation of the xylose-fermenting yeast *Scheffersomyces stipitis* to lignocellulosic hydrolysates

*Samuel Vega-Estévez, Andrew Armitage, Bruce S Dien, Patricia J Slininger and Alessia Buscaino*

**Content:**

Figure S1: AlphaFold predictions indicate no major structural differences between wild-type and mutant proteins in *sup-4*.

Figure S2: No major genomic differences between WT and *sup-4* strains.

Figure S3 Conservation of the NUDIX hydrolase Ysa1 across yeast species.

Supplementary Methods

**Figure S1. AlphaFold predictions indicate no major structural differences between wild-type and mutant proteins in *sup-4*.**
**(A)** AlphaFold-predicted structures of PICST_49608, PICST_62836, PICST_33084, and PICST_73970, the four proteins carrying missense substitutions in *sup-4*. Predicted domains are shown in different colors, and mutated residues are highlighted in green.
**(B)** Close-up views of the regions surrounding the mutated residues in wild-type (WT) and *sup-4* models. Side chains of the affected residues are shown in green. No differences in local secondary structure or overall folding are predicted between WT and mutant proteins, suggesting that these substitutions are unlikely to disrupt protein structure.

**Figure S2. No major genomic differences between WT and *sup-4* strains.**
Circos plot illustrating macrosynteny between the wild-type (WT; left) and *sup-4* (right) strains, with chromosome (Chr) numbers indicated.

**Figure S3. Conservation of the NUDIX hydrolase Ysa1 across yeast species.**
**(A)** Schematic representation of Ysa1 proteins from *S. stipitis*, *C. albicans*, and *S. cerevisiae*, including the conserved NUDIX hydrolase domain (green).
**(B)** Multiple sequence alignment of Ysa1 orthologs from *S. stipitis*, *C. albicans*, and *S. cerevisiae*. Conserved residues are highlighted in blue, and the conserved NUDIX motif is indicated by a green bar.

**Supplementary Methods**

***Bioinformatic Analysis***

Genome assembly and bioinformatic analyses are documented in the GitHub repository: https://github.com/harrisonlab/pichia/tree/master and were executed on HPC systems across NIAB East Malling Research, University of Kent and University of Greenwich. Job submission scripts via SunGridEngine are called within GitHub documentation but are documented within organisational private repositories. As such, the details of core run commands executed for each program are detailed below.

Genome assembly

Base-calling and demultiplexing were conducted with Albacore v2.3.3.

\# Albacore basecalling

read_fast5_basecaller.py --flowcell FLO-MIN106 --kit SQK-LSK108 --input raw_fast5_dir --recursive --worker_threads 24 --save_path output_dir --output_format fastq,fast5 --reads_per_fastq_batch 4000 --barcoding

Adapters and low-quality data were trimmed using the eautils package fastq-mcf 1.04.636 (https://expressionanalysis.github.io/ea-utils/).

\# Fastq-mcf adapter removal

fastq-mcf adapters.fa R1.fastq R2.fastq -o R1_trimmed.fastq -o R2_trimmed.fastq

For nanopore sequence data, adapter trimming was performed using Porechop v.0.1.0 (https://github.com/rrwick/Porechop).

\# Porechop adapter removal

porechop-runner.py -i reads.md5 -o reads_trimmed.fastq –threads 16 > trim_log.txt

Genome assembly was completed using long reads, with read correction performed with Canu (Koren et al. 2017) followed by assembly in SMARTdenovo github commit id 61cf13d (Li et al. 2009)

\# Canu read correction

canu -correct genomeSize=16m -p strain -d canu_out -nanopore-raw reads.fastq

canu -correct -useGrid=false -overlapper=mhap -utgReAlign=true -d canu_out -p prefix genomeSize=16m -nanopore-raw nanopore_reads.fq.gz

canu -trim -useGrid=false -overlapper=mhap -utgReAlign=true -d canu_out -p prefix genomeSize=16m -nanopore-corrected canu_out/strain.correctedReads.fasta.gz

\# SMARTdenovo assembly

smartdenovo corrected_reads.fasta.gz > assembly.fa

smartdenovo.pl -t corrected_reads.fasta.gz -p prefix > prefix.mak

make -f prefix.mak 2>&1 | tee prefix_run_log.txt

The draft assembly was corrected using the corrected nanopore reads through five rounds of Racon github commit 24e30a9 and then by raw fast5 files using 10 rounds of Nanopolish (Li and Durbin 2009). Finally, Illumina sequencing reads were used to polish the resulting assembly through 10 rounds of Pilon (Walker et al. 2014).

\# Racon polishing

racon reads.fastq alignments.sam assembly.fa > polished.fa

\# Nanopolish polishing

nanopolish index -d fast5_dir reads.fastq

nanopolish variants --consensus polished.fa -r reads.fastq -b alignments.bam -g assembly.fa

\# Pilon polishing

pilon --genome assembly.fa --frags illumina.bam --output pilon_out --fix all

After genome assembly, BUSCO (Simão et al. 2015) was run to assess the presence of evolutionarily conserved single-copy genes, using the Saccharomycetales_odb9 gene database (**Table S7**).

run_BUSCO.py -i assembly.fa -l saccharomycetales_odb9 -m geno -c 4

Assembly size and contiguity statistics were assessed using QUAST v4.5 (Gurevich et al. 2013).

quast.py --fast --fungus --gene-finding assembly.fa

Gene prediction and functional annotation

Gene prediction was performed using FunGap (v.1.0.1), with RNA-Seq transcript evidence provided through provision of unaligned and aligned illumina reads from S. stipitis NRRL Y-7124T NCBI accession SRR8420582 (BioSample SAMN09064163). RNA-Seq alignments were generated using STAR (v.2.7.4), following read-trimming of illumina sequence data. Gene model evidence was also provided through download and use of the proteome for reference strain Y-11545 NCBI accession GCA_000209165.1. Gene functional annotations generated using InterProScan (v5.42-78.0) and through BLASTp searches versus all proteins from the NCBI reference fungal genomes (downloaded 11 April 2020), retrieving the top-scoring blast hit with an E value of <1 × 10−30.

\# Star read alignment

STAR --runMode genomeGenerate --genomeDir genome-directory --genomeFastaFiles genome-directory/assembly.fa --genomeSAindexNbases 11

STAR --genomeDir genome-directory --outFileNamePrefix star_alignment

--readFilesCommand zcat --readFilesIn P.stipitis-Y-7124_F_trim.fq.gz P.stipitis-Y-7124_R_trim.fq.gz --outSAMtype BAM Unsorted --outSAMstrandField intronMotif

\# Sorting and indexing of aligned reads

samtools sort -o star_alignmentAligned.sortedByCoord.out.bam star_alignmentAligned.out.bam

samtools index star_alignmentAligned.sortedByCoord.out.bam

\# Fungap gene prediction

python fungap.py --output_dir out -1 P.stipitis-Y-7124_F_trim.fq.gz -2 P.stipitis-Y-7124_R_trim.fq.gz --genome_assembly assembly.fa --augustus_species pichia_stipitis --sister_proteome Pstipitisv2.FrozenGeneCatalog20080115.proteins.fasta --trans_bam alignedRNA.bam --busco_dataset “saccharomycetes_odb10” --translation_table 12

\# InterProScan functional annotation

interproscan.sh -goterms -iprlookup -dp -i proteins.fa

\# Blast vs refseq

blastp -query proteins.fa -db fungal-refseq.db -outfmt 6 -max_target_seqs 1 -max_hsps 1 -evalue 1e-30 -out prefix

Alignment, variant calling

A chromosome-level assembly was manually curated by identification of centromere sequences and overlapping regions between the contigs by BLASTN.

To unveil the presence of genome translocations, the assembled genomes were aligned against each other and against the parental NRRLY-7124 (Vega-Estévez et al. 2021) using the program Nucmer, inside Mummer v3.23 (Marçais et al. 2018).

\# Nucmer alignment

nucmer -t 8 -p prefix reference.fa query.fa

delta-filter -i 70 -l 5000 -r -q prefix.delta > filtered.delta

show-coords -c -l -b -T filtered.delta > coords.tsv

Minimap2 v0.2 was used for the alignment of the raw ONT reads against the genomes for subsequent study of the read coverage with SAMtools v1.3 (http://samtools.sourceforge.net) (Li 2018).

\# Minimap alignment of ONT reads

minimap2 -ax map-ont reference.fa reads.fastq > aligned.sam

\# Samtools processing of files

samtools view -bS aligned.sam > aligned.bam

samtools sort aligned.bam > aligned_sorted.bam

samtools index aligned_sorted.bam

\# Read coverage information was extracted

samtools depth -aa aligned_sorted.bam > depth.tsv

\# Median read coverage was determined using

cat depth.tsv | cut -f3 | sort -n | awk ' { a[i++]=$1; } END { print a[int(i/2)]; }'

Both type of alignments were plotted using Circos v0.69.4 and Rstudio ((http://www.R-project.org/).

SAMtools was also used for extracting, sorting and indexing of the ONT reads alignment over the genomes assembled, and bedtools v2.26.0 for the alignment transformation from bam to fasta format. The alignments in fasta were subsequently visualised with the software Integrative genome viewer v2.8.0 (IGV, from the Broad Institute) (Thorvaldsdottir et al. 2013).

\# Samtools

samtools view aligned_sorted.bam "contig_3:1082100-1082200" -h > 591_cen5_region_200Bp.bam

samtools sort -n 591_cen5_region_200Bp.bam > 591_cen5_region_200bp_sorted.bam

bedtools bamtofastq -i 591_cen5_region_200bp_sorted.bam -fq 591_cen5_region_200bp_sorted.bam.fq

The alignment of all the Illumina reads from the three strains was conducted with BWA-MEM (Burrows-Wheeler Alignment) version 0.7.15 (Li and Durbin 2009).

\# BWA read alignment

bwa index reference.fa

bwa mem -M -t 12 reference.fa R1.fastq R2.fastq | samtools view -bS - > sample.bam

\# Samtools sorting of alignment file

samtools sort sample.bam -o sample_sorted.bam

samtools index sample_sorted.bam

This was used for both the determination of the Illumina read coverage over the assembly and for the determination of variants (SNPs).

samtools depth -aa aligned.bam > depth.tsv

\# A custom python script was written to determine mean read coverage over 10kb sliding windows.

cov_by_window.py --cov depth.tsv > depth_window.tsv

The variant calling was conducted with the Genome Analysis Toolkit (GATK4) (Danecek et al. 2011) for all strains against the parental reference genome (GCA_016859295.1). Documentation of commands used in variant calling are documented in the GitHub file: https://github.com/harrisonlab/pichia/blob/master/popgen/SNP_calling.md

Effect upon gene models was assessed using SnpEff with a custom genome database generated from the parental reference genome and associated gene models.

\# Remove PCR and optical duplicates

java -jar picard.jar MarkDuplicates INPUT=sorted.bam OUTPUT=sorted_nodup.bam METRICS_FILE=sorted_nodup.txt REMOVE_DUPLICATES=TRUE ASSUME_SORTED=TRUE MAX_RECORDS_IN_RAM=500000000 MAX_FILE_HANDLES_FOR_READ_ENDS_MAP=1000 VALIDATION_STRINGENCY=LENIENT

\# Add group and sample name (Prefix)

java -jar picard.jar AddOrReplaceReadGroups INPUT=sorted_nodup.bam OUTPUT=sorted_nodup_rg.bam SORT_ORDER=coordinate CREATE_INDEX=true RGID=readgroupID RGSM=readgroupID RGPL=Illumina RGLB=library RGPU=barcode VALIDATION_STRINGENCY=LENIENT

\# Samtools indexing of generated bam file

samtools index sorted_nodup_rg.bam

\# Picard to prepare reference indexes required by GATK

java -jar picard.jar CreateSequenceDictionary R=assembly.fa O=assembly.dict

\# Samtools indexing of reference assembly

samtools faidx assembly.fa

\# GATK variant calling (note a -I was provided for each illumina dataset aligned against the reference assembly e.g. sup2 and sup4)

gatk HaplotypeCaller -R reference.fa -ploidy 1 -I sample1.bam -I sample2.bam -O output.vcf

\# hardfiltering of SNP calls using vcftools, generating outputs for indels and SNPs

vcffilter -f "QUAL > 30 & MQ > 40" raw.vcf | vcffilter -g "DP > 10 & GQ > 30" > filtered.vcf

vcftools --vcf filtered.vcf --max-missing 1.0 --remove-indels --recode --out final_no-indels

vcftools --vcf filtered.vcf --max-missing 1.0 ----keep-only-indels

--recode --out final_only-indels

perl vcf-stats filtered.vcf > stats.txt

\# Build a SnpEFF database

java -jar snpEff.jar build -gff3 -v databasename

java -jar snpEff.jar genome filtered.vcf > annotated.vcf

java -Xmx4g -jar $SnpEff/snpEff.jar -v -ud 0 databasename filtered.vcf > annotated.vcf

Annotated SNPs were partitioned into those with the following effects, based upon their respective annotations by SnpEFF SnpSift::

<effect>

genic: "(ANN[*].EFFECT has 'missense_variant') || (ANN[*].EFFECT has 'nonsense_variant') || (ANN[*].EFFECT has 'synonymous_variant') || (ANN[*].EFFECT has 'intron_variant') || (ANN[*].EFFECT has '5_prime_UTR_variant') || (ANN[*].EFFECT has '3_prime_UTR_variant')"

coding: "(ANN[0].EFFECT has 'missense_variant') || (ANN[0].EFFECT has 'nonsense_variant') || (ANN[0].EFFECT has 'synonymous_variant')"

non-synonymous: "(ANN[0].EFFECT has 'missense_variant') || (ANN[0].EFFECT has 'nonsense_variant')"

synonymous: "(ANN[0].EFFECT has 'synonymous_variant')"

java -jar SnpSift.jar filter <effect> annotated.vcf > filtered_by_effect.vcf

Identification of genomic features

The position of the centromeres, transposons and subtelomeric genes was determined using the online tool BLASTN with default settings. YSA1 sequences were aligned by Clustal-Wallis using Jalview v2.11.1.0 (Waterhouse et al. 2009).

Protein structure prediction and visualization.

Protein structural models were generated using AlphaFold2, as implemented in the AlphaFold Protein Structure Database, using default parameters. Predicted structures were visualized and compared using UCSF ChimeraX (Pettersen et al. 2021)

**REFERENCES**

Danecek P, Auton A, Abecasis G, Albers CA, Banks E, DePristo MA, Handsaker RE, Lunter G, Marth GT, Sherry ST et al. 2011. The variant call format and VCFtools. *Bioinformatics* **27**: 2156–2158.

Koren S, Walenz BP, Berlin K, Miller JR, Bergman NH, Phillippy AM. 2017. Canu: Scalable and accurate long-read assembly via adaptive κ-mer weighting and repeat separation. *Genome Research* **27**: 722–736.

Li H. 2018. Minimap2: pairwise alignment for nucleotide sequences. *Bioinformatics* **34**: 3094–3100.

Li H, Durbin R. 2009. Fast and accurate short read alignment with Burrows-Wheeler transform. *Bioinformatics* **25**: 1754–1760.

Li H, Handsaker B, Wysoker A, Fennell T, Ruan J, Homer N, Marth G, Abecasis G, Durbin R. 2009. The Sequence Alignment/Map format and SAMtools. *Bioinformatics* **25**: 2078–2079.

Marçais G, Delcher AL, Phillippy AM, Coston R, Salzberg SL, Zimin A. 2018. MUMmer4: A fast and versatile genome alignment system. *PLOS Computational Biology* **14**: e1005944–e1005944.

Pettersen EF, Goddard TD, Huang CC, Meng EC, Couch GS, Croll TI, Morris JH, Ferrin TE. 2021. UCSF ChimeraX: Structure visualization for researchers, educators, and developers. *Protein Sci* **30**: 70–82.

Simão FA, Waterhouse RM, Ioannidis P, Kriventseva EV, Zdobnov EM. 2015. BUSCO: Assessing genome assembly and annotation completeness with single-copy orthologs. *Bioinformatics* **31**: 3210–3212.

Thorvaldsdottir H, Robinson JT, Mesirov JP. 2013. Integrative Genomics Viewer (IGV): high-performance genomics data visualization and exploration. *Brief Bioinform* **14**: 178–192.

Vega-Estévez S, Armitage A, Bates HJ, Harrison RJ, Buscaino A. 2021. The Genome of the CTG(Ser1) Yeast Scheffersomyces stipitis Is Plastic. *mBio* doi:10.1128/MBIO.01871-21.

Walker BJ, Abeel T, Shea T, Priest M, Abouelliel A, Sakthikumar S, Cuomo CA, Zeng Q, Wortman J, Young SK et al. 2014. Pilon: An Integrated Tool for Comprehensive Microbial Variant Detection and Genome Assembly Improvement. *PLoS ONE* **9**: e112963–e112963.

Waterhouse AM, Procter JB, Martin DMA, Clamp M, Barton GJ. 2009. Jalview Version 2--a multiple sequence alignment editor and analysis workbench. *Bioinformatics (Oxford, England)* **25**: 1189–1191.
